# The tumor microenvironment promotes cancer progression and cell migration

**DOI:** 10.18632/oncotarget.14155

**Published:** 2016-12-24

**Authors:** Viviana Salvatore, Gabriella Teti, Stefano Focaroli, Maria Carla Mazzotti, Antonio Mazzotti, Mirella Falconi

**Affiliations:** ^1^ DIBINEM, University of Bologna Department of Biomedical and Neuromotor Sciences, 40126 Bologna, Italy; ^2^ DIMEC, University of Bologna, Department of Medical and Surgical Sciences, 40126 Bologna, Italy; ^3^ Rizzoli Orthopaedhic Institute, 40136 Bologna, Italy

**Keywords:** tumor microenvironment, osteosarcoma, human fibroblasts, co-culture, tumor stroma

## Abstract

The tumor microenvironment contributes to cancer progression, in part through interactions between tumor and normal stromal cells. This study analyzed morphological and molecular changes induced in co-cultured human fibroblasts (HFs) and the MG-63 osteosarcoma cell line. Co-cultured cell monolayers were morphologically analyzed using high resolution scanning electron microscopy (HR-SEM), and trans-well assays were performed to assess cell migration and invasion. Proteins involved in inflammatory responses, cancer cell invasion, and angiogenesis were assessed using western blotting. HR-SEM showed progressive spatial orientation loss by fibroblasts in contact with MG-63s, while MG-63s proliferated rapidly and invaded HF space. Trans-well assays showed enhanced MG-63 migration in the presence of HFs. IL-6 expression was increased in co-cultured HFs, possibly stimulated by TNF-α. HFs do not normally express YKL-40 but did so in co-culture. Band densitometric analyses showed that increasing YKL-40 expression was followed by VEGF overexpression, especially in MG-63s. Finally, our results confirmed fibroblasts as the main matrix metalloproteinase source in this tumor microenvironment. Our study sheds new light on how tumor-stroma interactions promote tumor development and progression, and may support identification of novel anti-cancer therapeutics.

## INTRODUCTION

Cancers can adapt to different environmental conditions, changing cellular morphologies and genetic characteristics in order to survive [1]. Cancer cells are responsible for activation of nearby stromal cells, including fibroblasts, endothelial cells and macrophages [2]. Activated stromal cells reorganize connective tissue structure and composition by releasing chemotactic cytokines and growth factors, and depositing extracellular matrix (ECM) components [3]. This enhances tumor growth and invasion and promotes chemotherapy resistance [3, 4]. The aim of this work was to simulate the establishment of a tumor microenvironment between the osteosarcoma cell line, MG-63, and human fibroblasts (HFs) grown in co-culture. We showed that tumor and normal cell coexistence leads to morphological and molecular changes in both cell lines. Our analyses focused on some of the key factors involved in tumor induction and their roles in tumor angiogenesis, invasion, metastasis, and ECM composition.

### TNF alpha

Tumor necrosis factor alpha (TNF-α), a member of the TNF/TNFR cytokine superfamily, is assocated with chronic inflammation and the development of cancer. TNF-α expression within the tumor microenvironment promotes tumor cell invasion, migration and metastasis in several cancers. It partially activates the nuclear factor kappa-light-chain-enhancer of activated B cells (NF-κB) and the cytokine network in human cancer cells. It is also central to interactions between tumor cells and macrophages, promoting malignant cell invasion [5, 6]. TNF-α is produced at low picogram levels in the tumor microenvironment, whether by tumor or stromal cells [6]. Increased serum TNF-α concentrations are observed in several cancers, despite tumor cells producing only small amounts of the molecule [6]. TNF-α may directly damage DNA, mediate tumor and stromal cell interactions, inhibit apoptosis, promote cell proliferation, and induce expression of tumor development-promoting molecules [6]. In established tumors, TNF-α contributes to the maintenance of a pro-inflammatory environment [6].

### IL-6

Interleukin 6 (IL-6) is a multifunctional inflammatory cytokine involved in various biologic processes, including dysimmune diseases and cancers [7]. It orchestrates innate and adaptive immunity, mediates chronic inflammation and autoimmunity, and is a key cytokine linking chronic inflammation to cancer development [8]. Like TNF-α, IL-6 facilitates tumor development by promoting the conversion of non-cancer cells into tumor stem cells *in vitro* via Oct4 gene upregulation expression through IL-6R/JAK/STAT3 signaling [8]. Elevated IL-6 levels in human serum are associated with increased cancer risk. Thus, IL-6 is considered a prognostic marker and current anti-cancer therapies already target IL-6 activity [7].

### YKL-40

YKL-40, also known as chitinase-3-like protein 1, is a chitinase-like glycoprotein that lacks chitinase activity due to active site mutations. It interacts with glycosaminoglycans, including heparin and hyaluronan, and binds collagen types I–III. It suppresses E-cadherin, but increases matrix metalloproteinase-9 (MMP-9) expression and cell motility, which are each essential for tumor cell invasion [9]. YKL-40 purified from MG-63 cells induces fibroblast morphologic transformations near the tumor site, secretion of MMPs, and neovascularization [4], promoting cancer cell invasion and destruction of the stroma [10]. Serum YKL-40 levels are elevated in a variety of chronic inflammatory diseases, suggesting that its pathologic function involves ECM remodeling. Its expression is stimulated by cytokines, including IL-13, IL-6 and IL-1β [9].

### VEGF

Vascular endothelial growth factor (VEGF) is a homodimeric glycoprotein that mediates tumor angiogenesis. VEGF is upregulated by oncogenes, growth factors, and hypoxia. VEGF also supports tumor growth by protecting tumor neovasculature against apoptosis, through induction of the anti-apoptotic factors, Bcl-2 and survivin [11]. Additionally, VEGF induces secretion and activation of enzymes involved in ECM degradation, such as plasminogen activator and MMP-1, allowing for further blood vessel development [12]. YKL-40 is a potent angiogenesis inducer, and has been investigated in several types of cancer. Faibish, *et al*. found that YKL-40 up-regulates VEGF production in the glioblastoma cell line, U87, and demonstrated that monoclonal antibodies targeting YKL-40 activity are a promising strategy for treating advanced tumors [13].

### MMPs and MMP-9

During tumor cell invasion, matrix metallo-proteinases digest various ECM components, including proteoglycans, collagen, laminin, fibronectin, elastin, and vitronectin [14]. MMPs promote cancer cell invasion, migration, and metastasis [15]. The gelatinases, MMP-2 and MMP-9, degrade type IV collagen and gelatin, the two main ECM and basement membrane structural proteins. MMP-9 overexpression is associated with enhanced tumor cell invasion and overall aggression [16, 17].

## RESULTS

### High resolution scanning electron microscopy (HR-SEM)

**Figure 1:**
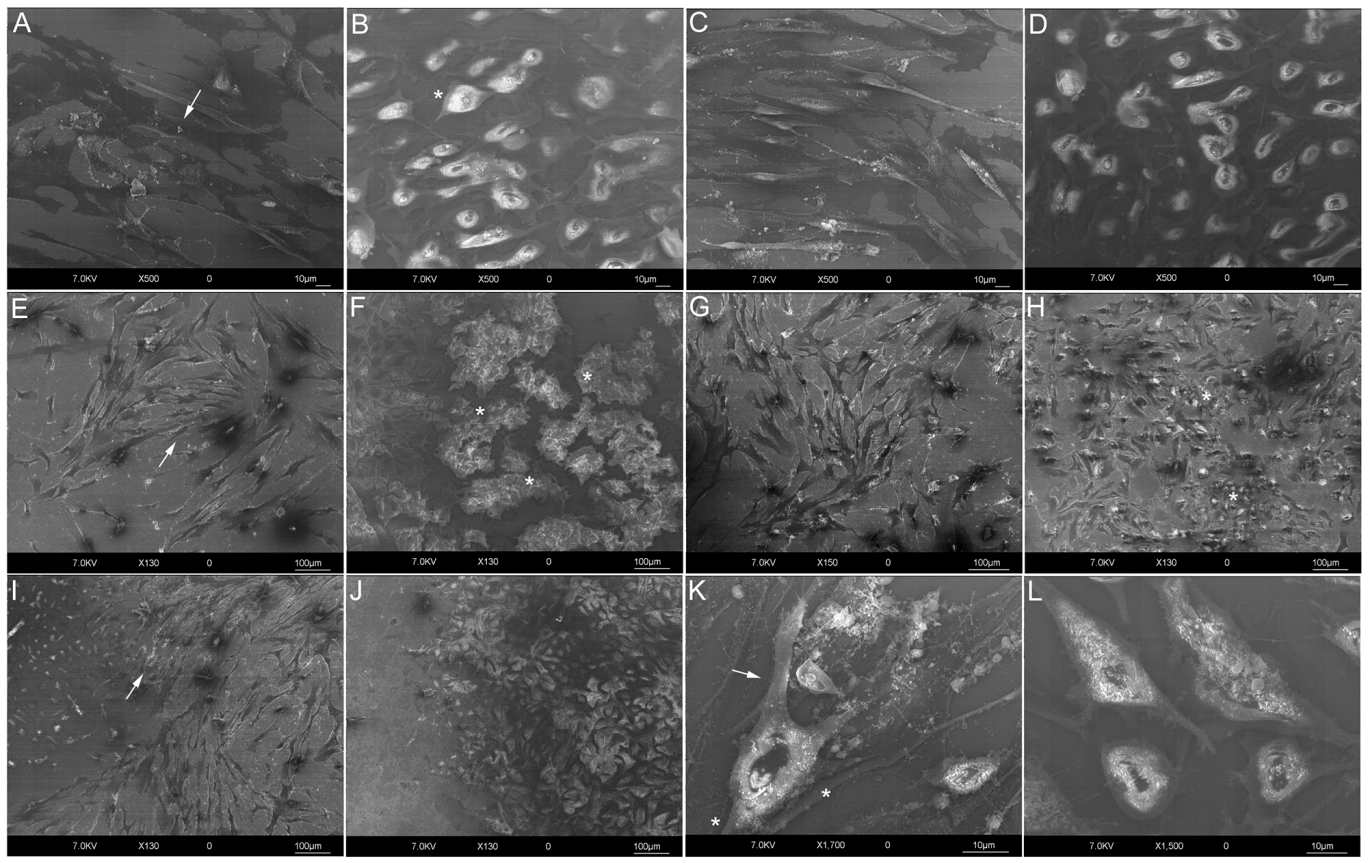
HR-SEM analyses HF control at 24 h (Bar: 10 μm) **A**. Flat and elongated morphology (arrow). MG-63 control at 24 h (Bar: 10 μm) **B**. Polygonal morphology (star). HFs **C**. and MG-63s **D**. after 24 h of co-culture (Bar: 10 μm). HFs **E**. and MG-63s **F**. after 48 h of co-culture. Loss of fibroblast orientation (E., arrow); reduced MG-63 proliferation (Bar: 100 μm) and formation of clusters (F., stars). HFs **G**. and MG-63s **H**. after 72 h of co-culture (Bar: 100 μm). MG-63 invasion of HF space (H., stars). HFs **I**. and MG-63s **J**. after 96 h of co-culture (Bar: 100 μm). Overlap of MG-63s with HFs (**I**., arrow). Detail of MG-63s at 96 h of co-culture **K**. Pseudopod protrusions (stars) and pre-uropod region (arrow) (Bar: 10 μm). Detail of MG-63 control at 96 h (Bar: 10 μm) **L**.

HR-SEM images depicted a clearly identifiable morphology for the control samples from both human fibroblast (HF) and MG-63 cells. After 24 h, HFs alone exhibited a fibroblastic morphology with an elongated cell body (Figure 1A, arrow) and MG-63s were polygonal (Figure 1B, star). In co-cultures, HFs at 48 h appeared more disordered (Figure 1E, arrow). After quickly reaching confluence and forming clusters (Figure 1F, stars), MG-63 cells moved toward HFs, invading their space by 72 h (Figure 1H, stars) and overlapping them at 96 h (Figure 1I). Co-cultured MG-63 morphology changed at 96 h (Figure 1K) compared to controls (Figure 1K–1L), showing some pseudopod protrusions and a pre-uropod region.

### Cell migration

**Figure 2:**
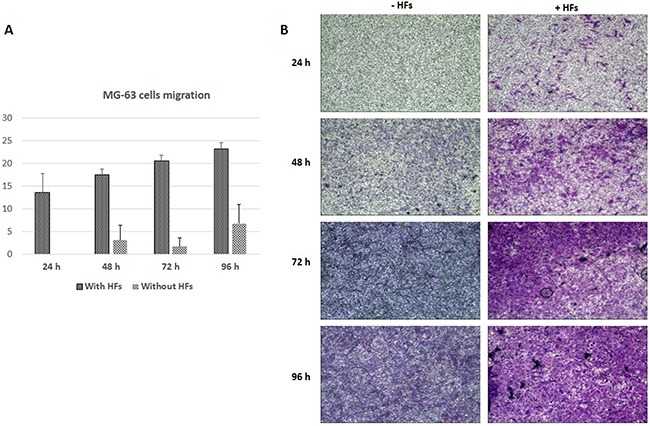
Cell migration assay MG-63 cell migration (%) **A**. * represents a difference between MG-63 cells cultured with and without HFs, P<0.05. Giemsa staining of migrating MG-63 cells, with or without HFs **B**.

MG-63 cells cultured without HFs reached maximum migration levels of about 7% at 96 h (Figure 2A). When co-cultured with HFs, MG-63 migration gradually increased up to a maximum of 24% at 96 h (Figure 2A–2B).

### Western blot and densitometric analyses

**Figure 3:**
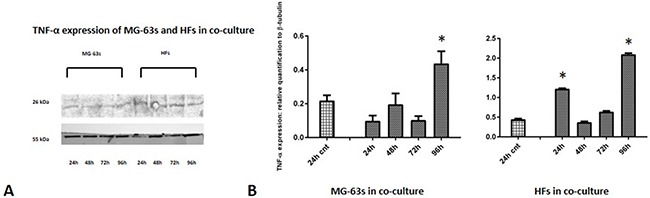
Western blot for TNF-α Densitometric analysis **A**. TNF-α expression in co-cultured HFs and MG-63 cells **B**. * represents a difference between co-cultured cells and HF or MG-63 cells alone. P<0.05.

**Figure 4:**
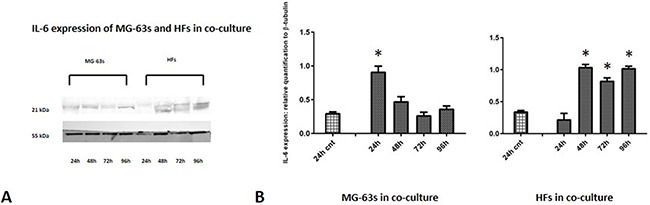
Western blot for IL-6 Densitometric analysis **A**. IL-6 expression in co-cultured HFs and MG-63 cells **B**. * represents a difference between co-cultured cells and HF or MG-63 cells alone. P<0.05.

**Figure 5:**
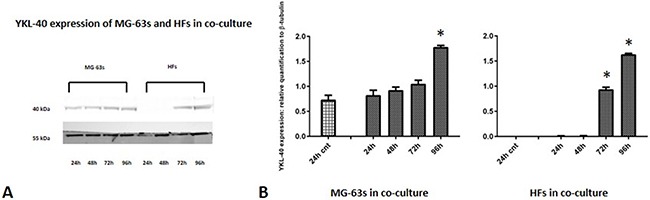
Western blot for YKL-40 Densitometric analysis **A**. YKL-40 expression in co-cultured HFs and MG-63 cells **B**. * represents a difference between co-cultured cells and HF or MG-63 cells alone. P<0.05.

**Figure 6:**
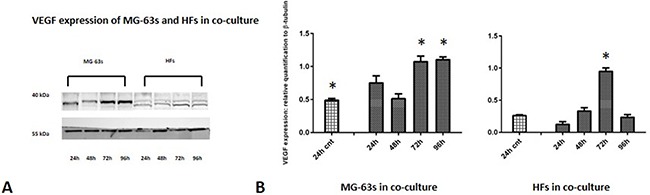
Western blot for VEGF Densitometric analysis **A**. VEGF expression in co-cultured HFs and MG-63 cells **B**. * represents a difference between co-cultured cells and HF or MG-63 cells alone. P<0.05.

**Figure 7:**
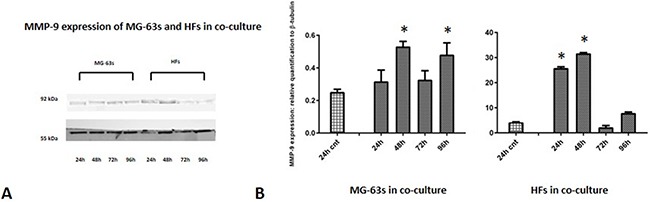
Western blot for MMP-9 Densitometric analysis **A**. MMP-9 expression in co-cultured HFs and MG-63 cells **B**. * represents a difference between co-cultured cells and HF or MG-63 cells alone. P<0.05.

Densitometric analysis results showed lower TNF-α levels in co-cultured MG-63s than in HFs. In both cell lines, TNF-α bands reached maximum intensities after 96 h (Figure 3). IL-6 expression in MG-63s decreased after 24 h and remained stable. In HFs, IL-6 increased at 48 h, then remained constant (Figure 4). YKL-40 expression in MG-63s increased until 96 h, while in HFs, YKL-40 was not expressed until 48 h. Expression remained high in HFs at 72 and 96 h (Figure 5). MG-63s exhibited higher VEGF levels compared to HFs, with an increase at 72 and 96 h. VEGF expression was low in HFs at 24 and 48 h, reaching maximum levels at 72 h and decreasing again at 96 h (Figure 6). Low MMP-9 levels were detected in MG-63 cells, with maximal levels at 72 and 96 h. Very high MMP-9 expression was observed in HFs after 24 and 48 h, with a decrease after 72 h (Figure 7).

## DISCUSSION

The tumor microenvironment actively contributes to cancer progression, and may direct genetic and epigenetic changes within cells involved. Recent studies showed that biochemical and physical signals could profoundly alter both tumor and nearby normal cells. New *in vitro* cell culture techniques allow for analysis of multiple physical and molecular culture parameters, including ECM pore size, fiber alignment, cell proliferation, and metastatic potential, clarifying how these components drive tumor progression. Stromal cells, such as fibroblasts or immune cells, can work in concert with cancer cells during tumor development to promote tumor progression via ECM modifications [18]. While stromal cells are not malignant, interactions with each other, the tumor microenvironment, and with cancer cells can induce phenotypic and functional changes [19]. Normal-malignant cell contact and crosstalk further drives the cancer phenotype [19, 20].

The aim of this work was to analyze the morphological and molecular changes that occour in co-cultured human fibroblasts and osteosarcoma cells during a simulation of the tumor microenvironment. We observed direct stimulation of tumor cell growth, proliferation, and migration by growth factors and chemokines originating from both fibroblasts and cancer cells. A morphological HR-SEM analysis was performed on co-cultured MG-63 and HF cells grown in monolayers and on the same cells cultured alone. While fibroblasts seeded in co-culture seemed to lose their orderly organization patterns early, MG-63s exhibited characteristics of rapidly growing cells, reaching confluence faster than HFs. MG-63 cells formed clusters, intruding into HF space and almost overlapping them. Invasive malignant cells in co-culture appeared to change their shapes compared to controls, showing cytoskeletal polarization and protrusions. We believe that MG-63 cells in our study were in an early migration stage, showing relatively few specialized structures, like pseudopod protrusions opposite a “pre-uropod” region, as described by Friedl and Alexander [21, 22].

A cell migration assay in a trans-well system was performed to futher assess MG-63 cell migration in co-culture with HFs. MG-63s showed little or no migration (7% at 96 h) when cultured alone, but when co-cultured with HFs, MG-63 cell migration gradually increased throughout the experiment (24% at 96 h). We hypothesized that MG-63s responded to HF-secreted paracrine signals and growth factors that acted as chemioattractants. Tumor cell dissemination is thought to occur in part via chemotaxis [23]. Directional migration toward a chemokine source is observed *in vitro* and *in vivo* for cells of the tumor microenvironment, leading to cancer cell invasion, inflammatory responses, and angiogenesis [23]. Some of the key factors involved in these processes were therefore analyzed in our study, including TNF-α, IL-6, YKL-40, VEGF and MMP-9.

TNF-α and IL-6 are central mediators of inflammation, and contribute to the maintenance of a pro-inflammatory environment. TNF-α is produced at low levels in the tumor microenvironment by tumor or stromal cells, or probably both [6], and perpetuates chronic inflammation and autoimmunity together with IL-6, a key cytokine linking chronic inflammation to cancer. Our western blotting results showed that TNF-α levels were lower in MG-63 cells than in HFs, and densitometric analysis revealed that in both cell types, the signal increased through 96 h of co-culture. TNF-α expression induces rapid IL-6 activation in tissue affected by inflammation, which explains the increase in IL-6 expression in fibroblasts under co-culture conditions. HFs co-cultured with MG-63s showed increased IL-6 expression after 48 h [24].

As TNF-α and IL-6 are major regulators of acute inflammation, and IL-6 appears to promote YKL-40 expression [25], YKL-40 overexpression is associated with increased inflammation. YKL-40 is a glycoprotein normally expressed *in vitro* by MG-63 cells, potentially promoting the angiogenic switch and remodelling of the ECM, as hypothesized in our previous work [4]. YKL-40 western blotting results confirmed our prior results: HFs that do not normally express YKL-40 *in vitro* begin to do so at 72 h when co-cultured with MG-63 cells, reaching the same maximum signal levels as MG-63s at 96 h. Moreover, band densitometric analyses showed that increasing YKL-40 expression was followed by VEGF overexpression, especially in MG-63s at 72 and 96 h, confirming that YKL-40 promotes angiogenesis [13].

Finally, our results confirmed fibroblasts as the main source for MMPs in the tumor microenvironment. MMPs promote tumor cell invasion, migration, and metastasis [15], and originate from both tumor and benign cells, such as fibroblasts [26]. In part, this allows the so-called tumor-associated fibroblasts to stimulate tumor cell motility and invasion. MG-63 cells showed weak MMP-9 expression compared to HFs, which exhibited elevated protein levels after only 24 h. This suggests that the tumor microenviroment modifies the stroma by upregulating the matrix metalloproteinases, which are responsible for ECM disruption, cell migration and tumor metastasis. The net balance between metalloproteinases and their specific endogenous tissue inhibitors (TIMPs) activities determines the proteolytic potential of tumors [27]. Decreases in TIMP levels are correlated with tumorigenesis, and in our model we presumed that MMP-9 secreted by fibroblasts was not balanced by its inhibitor, TIMP-1. However, elevated TIMP-1 expression reportedly promoted cancer cell proliferation and invasion and correlated with progression and unfavourable prognosis in certain tumor types [27]. Analysis of TIMP-1 expression and activity by immunoblotting and zymography are still needed for a better understanding of our model, along with investigations of cellular responses to MMP/TIMP ratios.

Inhibition of MMP expression and/or activity may effectively prevent tumor invasion and metastasis. Recent strategies for blocking MMP gene transcription target extracellular factors, signal-transduction pathways, or nuclear factors that activate MMP gene expression [28]. The use of ribozymes or antisense constructs downregulates MMP production and/or TIMP1 expression [28]. Although there are many pathways toward blocking MMP production, none have been translated into the clinical, due in part to the diversity of MMP-regulating molecules, and the opposing effects of different factors on the expression of different MMPs [28]. These observations highlight the need for novel MMP inhibition strategies. A decade ago, the FDA approved Avastin as the first drug specifically targeting the tumor microenvironment in cancer [29]. Since this milestone, preclinical development and clinical testing of tumor microenvironment-targeting agents has increased remarkably, and several such agents are already standard treatments in patients with specific cancers [29]

As shown by the large number of chemotherapeutics approved by the FDA, strategies targeting the tumor vasculature also appear successful [29]. Furthermore, strategies inhibiting protumorigenic inflammatory responses are rapidly being developed with agents targeting pathways activated in tumor and stromal cells [29, 30]. Assessing new tumor microenvironment-targeting agents in combination with radiotherapy and other chemotherapeutics may help avert tumor resistance to treatment. Additionally, because the pro- or anti-tumorigenic functions of the tumor microenvironment change during cancer progression, therapeutic agents must be assessed for effectiveness at various disease stages in specific cancers [29]. Reliable biomarkers that indicate the type of tumor stroma present are also needed for improved patient outcomes [29].

In conclusion, our results emphasize the microenvironment as an intrinsic aspect of the tumor itself. As the ECM differs between cancer types, *in vitro* systems should be adapted to simulate the native stromal environment. Experiments combining cancer and stromal cells are necessary to enhance understanding of such interactions during cancer cell invasion and metastasis [1, 20] and may lead to the identification of novel anti-cancer therapeutic strategies.

## MATERIALS AND METHODS

### Primary human fibrobast cultures

Human gingival tissues were collected from the mid-third of the roots of teeth extracted for orthodontic reasons, with informed patient consent. After several washes in PBS, tissues were cut into small pieces and placed into culture dishes with 1 mL of Dulbecco's Modified Essential Medium (DMEM) (Invitrogen, Carlsbad, CA, USA) supplemented with 10% (v/v) fetal bovine serum (FBS), penicillin (100 mg/mL), and streptomycin (10 mg/mL). Culture medium was changed twice per week. Subconfluent (70–80%) HFs were detached from culture dishes using 0.05% trypsin/EDTA (Gibco, Grand Island, NE, USA), washed, and placed into T75 flasks. Cells were then cultured at 37°C in a humidified atmosphere with 5% CO_2_. Cells from passages 3 to 10 were used for experiments.

### MG-63 cell culture

The MG-63 osteosarcoma cell line was purchased from ATCC (Manassas, VA, USA; CRL-1427) and cultivated in DMEM supplemented with 10% (v/v) FBS, penicillin (100 mg/mL), and streptomycin (10 mg/mL). Cells were cultured in T25 flasks (Nunc, Waltham, MA, USA) in a humidified incubator at 37°C with 5% CO_2_. For passaging, cells were detached with trypsin/EDTA and replated.

### HF and MG-63 cell co-cultures

We performed a transwell co-culture, with HF and MG-63 cells seeded at 2.5×10^5^ cells/mL each. HFs were pre-cultured in 6-well plates, while MG-63 cells were seeded in 6-well transwell inserts (Transwell® permeable support, Corning Life Sciences). After 24 h, each support was put in the 6-well plates and co-cultured in DMEM supplemented with 10% (v/v) FBS, penicillin (100 mg/mL), and streptomycin (10 mg/mL) for 24, 48, 72, or 96 h. HFs and MG-63 cells were collected and analyzed at the same time points.

### HR-SEM analysis

HF and MG-63 cells were seeded on sterile silica supports at 10×10^3^ cells/ml and co-cultured for 24, 48, 72 or 96 h in petri dishes, in DMEM supplemented with 10% (v/v) FBS, penicillin (100 mg/mL), and streptomycin (10 mg/mL). The same cell types seeded separately on silica supports at the same density were used as control. At each collection point, silica supports were washed in sodium cacodylate buffer 0.15 M, fixed with 2.5% glutaraldehyde in 0.1 M sodium cacodylate buffer (Sigma Aldrich, St. Louis, Missouri, USA) for 2 h at 4°C, and post-fixed with 1% OsO_4_ (Società Italiana Chimici, Roma, Italia) in 0.1 M sodium cacodylate buffer for 1 h at room temperature. After several washes in 0.15 M sodium cacodylate buffer, samples were dehydrated in increasingly concentrated alcohol solutions and then processed by critical point drying (030 Critical Point Dryer, Bal-Tec, Leica Microsystems GmbH, Wetzlar, Germany). Samples were metallized by a thin layer of platinum carbon (Bal-Tec, Leica Microsystems GmbH) and observed by HR-SEM (JSM 890, Jeol Company, Tokyo, Japan) with an accelerated voltage of 10 kV and 1×10−11 mA.

### Cell migration assay

A transwell system (Transwell® permeable support, Corning Life Sciences, membrane pore size: 3.0 μm) was used to analyze cell migration. MG-63 cells were harvested and suspended in serum-free DMEM at 2.5×10^5^ cells/mL. The suspension was seeded into the upper chamber of the transwell system. HFs were harvested and suspended in serum-free DMEM at the same concentration and seeded in the bottom wells. As a control, MG-63s were suspended in DMEM serum free at the same concentration and seeded into the upper wells, while in the bottom wells 2 mL of DMEM supplemented with 10% (v/v) FBS, penicillin (100 mg/mL), and streptomycin (10 mg/mL) were added. The wells were incubated at 37°C for 24, 48, 72 and 96 h. At each collection point, medium was removed from the inserts, inserts were washed twice in PBS, and cells were fixed in formaldheyde (3.7% in PBS) for 2 min at room temperature. The formaldheyde was removed and cells were washed twice in PBS and permeabilized in 100% methanol at room temperature for 20 min. The methanol was removed and cells were washed twice in PBS and stained with Giemsa (Sigma Aldrich) at room temperature for 15 min. Cells were then washed twice in PBS and non-migrated cells were removed with cotton swabs. Migrated cells were counted under a phase contrast microscope (Motic AE21, Seneco Srl, Milano, Italy). Membranes were photographed in triplicate and images were recorded using Visicam 3.0 and analyzed by VisiCam Image Analyzer software, version 6.1.3.3 (VWR International Srl, Milano, Italy). The stained area was quantifyed using ImageJ software (National Institutes of Health, Bethesda, MD) and expressed as a percentage (stained areas versus non-stained areas).

### Western blotting and densitometric analysis

At each experimental time point, cell pellets were lysed for 30 min using RIPA buffer (Invitrogen, Life Technologies, Monza Italy) supplemented with a protease inhibitor cocktail (Sigma Aldrich), 1 mM PMSF and 0.15% β-mercaptoethanol (Fluka, Sigma Aldrich). Samples were centrifuged at 14,000 rpm for 10 min at 4°C, and total protein concentrations were measured using Bradford reagent (Sigma Aldrich). Twenty μg of total protein was resolved on 12% SDS-PAGE Bolt® Bis-Tris Plus MES gels (Invitrogen). Proteins were transferred to a nitrocellulose membrane (GE Healthcare Europe GmbH), blocked with nonfat dry milk (Sigma Aldrich) for 30 min at room temperature, and immunolabeled with a primary antibody (anti-TNF-α, IL-6, YKL- 40, VEGF, and MMP-9, with β-tubulin as endogenous control) diluted in TBS pH 7.5 at 4°C overnight. Bands were visualized using an ECL Advanced TM Western blotting detection kit (GE Healthcare Europe GmbH) and images were recorded with a Kodak digital image station (Eastman Kodak, Rochester, NY, USA). Band densitometric analysis was performed using Image J software, and band intensities were corrected for equal β-tubulin loading. Intensities are provided relative to the intensities of controls. Densitometry data are represented as means ± standard deviations (SD) of three independent experiments.

### Statistical analysis

Statistical analysis was carried out using GraphPad Prism 5.0 software (San Diego, CA) via analysis of variance (ANOVA) and the Dunnet's multiple comparison test. P<0.05 was considered statistically significant.

## Abbreviations

HFs: Human fibroblasts
ECM: Extracellular matrix
TNF alpha: Tumor necrosis factor alpha
IL-6: Interleukin 6
YKL-40: Human cartilage glycoprotein–39
MMP-9: Matrix metalloprotease 9
MMPs: Matrix metalloproteinases
VEGF: Vascular Endothelial Growth Factor.
